# Effect of dry hydrogen peroxide on *Candida auris* environmental contamination

**DOI:** 10.1017/ash.2023.316

**Published:** 2023-09-29

**Authors:** Jennifer Sanguinet, Gerard Marshall, Julia Moody, Kenneth Sands

## Abstract

**Background:** *Candida auris* is an emerging pathogen that exhibits broad antimicrobial resistance and causes highly morbid infections. Prolonged survival on surfaces has been demonstrated, and standard disinfectants may not achieve adequate disinfection. Persistent patient colonization and constant environmental recontamination poses an infection risk that may be mitigated by no touch disinfection systems. We evaluated the efficacy of continuous dry hydrogen peroxide (DHP) exposure on *C. auris* environmental contamination. **Methods:** The study was conducted in a large tertiary-care center where multiple patients were identified as either infected or colonized with *C. auris*. DHP-emitting systems were installed in the ventilation systems dedicated to the adult burn intensive care and children’s cardiac intensive care units. Composite surface samples were collected in a sample of patient rooms and shared clinical workspaces among units with current *C. auris* patients, before and after installation of the DHP system, and from areas with and without exposure to DHP. The samples included “high touch” surfaces near the patient, the general area of the patient room, shared medical equipment for the unit, shared staff work areas, and equipment dedicated to individual staff members (Table 1). Presence of *C. auris* was determined by polymerase chain reaction (PCR). Association between DHP exposure and *C. auris* contamination was determined using the Fisher exact test. **Results:** In the presence of *C. auris* patients, 5 baseline samples per unit were taken before DHP was installed, and then 5 samples per unit were taken on days 7, 14, and 28 after installation. Prior to initiation of DHP, 7 (70%) of 10 samples were PCR positive for *C. auris*. After DHP installation, a statistically significant decrease to 5 (16.7%) of 30 samples (*P* <.05) was observed. In total, 20 samples (5 before installation and 15 after installation) were collected from units without DHP on the same days. At baseline, 2 (40%) of 5 samples were PCR positive for *C. auris*. During subsequent periods, 4 (27%) 15 samples were positive (*P* = .66). No adverse effects were reported by patients, visitors, or personnel in association with the operation of the DHP systems. **Conclusions:** These findings suggest that DHP is effective in reducing surface *C. auris* contamination in a variety of patient and healthcare worker surfaces.

**Figure:**
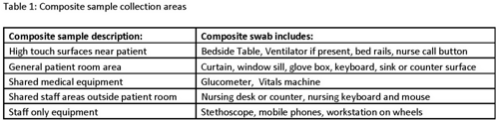

**Disclosures:** None

